# Locus Coeruleus MR Measured Signal Intensity in Fibromyalgia Relative to Healthy Controls

**DOI:** 10.1002/ejp.70173

**Published:** 2025-11-17

**Authors:** Marilena M. DeMayo, Clifford M. Cassidy, Cheryl McCreary, Tuan Trang, Ashley D. Harris, Alexander McGirr

**Affiliations:** ^1^ Department of Psychiatry University of Calgary Calgary Alberta Canada; ^2^ Hotchkiss Brain Institute University of Calgary Calgary Alberta Canada; ^3^ Mathison Centre for Mental Health Research and Education Calgary Alberta Canada; ^4^ Department of Radiology University of Calgary Calgary Alberta Canada; ^5^ Alberta Children's Hospital Research Institute University of Calgary Calgary Alberta Canada; ^6^ Department of Psychiatry Stony Brook University Stony Brook New York USA; ^7^ Department of Clinical Neuroscience University of Calgary Calgary Alberta Canada; ^8^ Department of Physiology and Pharmacology University of Calgary Calgary Alberta Canada; ^9^ Faculty of Veterinary Medicine University of Calgary Calgary Alberta Canada

**Keywords:** fibromyalgia, locus coeruleus, magnetic resonance, neuromelanin, noradrenaline

## Abstract

**Background:**

Fibromyalgia is a chronic pain condition without an established aetiology. However, noradrenergic dysfunction is a possible mechanism to explain the constellation of symptoms associated with fibromyalgia. Noradrenaline synthesis in the locus coeruleus (LC) results in a paramagnetic by‐product, neuromelanin. Recently, a magnetic resonance imaging sequence sensitive to neuromelanin has been used to assay LC signal intensity, a proxy for noradrenergic system function. Here, we use MR imaging to investigate the noradrenergic‐locus coeruleus system in participants with fibromyalgia and healthy controls.

**Methods:**

Forty‐six participants with fibromyalgia and 41 healthy controls were recruited for a cross‐sectional characterisation of LC signal intensity at 3 T, quantified from a 2D gradient echo acquisition. Participants completed the Revised Fibromyalgia Impact Questionnaire, as well as measures of anxiety, depression, sleep and the THINC‐it cognitive battery.

**Results:**

An independent groups *t*‐test revealed no differences in LC signal intensity between participants with fibromyalgia and healthy controls. For the participants with fibromyalgia, partial correlations accounting for age showed no association between LC signal intensity and fibromyalgia history, fibromyalgia symptom severity, anxiety, depression, insomnia or cognitive performance. Almost 90% of participants with fibromyalgia had been exposed to medications targeting noradrenergic function complicating the interpretation of these findings.

**Conclusions:**

LC signal intensity as measured by MR did not distinguish participants with fibromyalgia and healthy controls, nor was it associated with core fibromyalgia pain symptoms or associated symptoms. Dynamic measures of noradrenergic function may be required to understand noradrenergic contributions to fibromyalgia.

**Significance Statement:**

This study is the first report using MR measured signal intensity of the LC to examine noradrenergic function in participants with fibromyalgia. There was no difference in signal intensity when comparing patients to controls, nor did it associate with any symptoms or associated features of fibromyalgia. This suggests that lifetime noradrenergic function may not distinguish fibromyalgia.

Fibromyalgia is a chronic pain condition that affects approximately 2%–3% of the population (McNally et al. 2006). It is characterised by ongoing, widespread pain, along with accompanying symptoms such as anxiety, mood, disrupted sleep, and cognitive difficulties (Hauser et al. 2015). The pain experienced in fibromyalgia is described as centralised pain, where the pain is not a result of peripheral damage or inflammation; rather it appears driven by central mechanisms (Sluka and Clauw 2016). The mechanisms underlying this pain are not established.

A hypothesised mechanism for centralised pain in fibromyalgia is noradrenergic dysfunction (Clauw 2009), with evidence that patients with fibromyalgia have lower levels of cerebrospinal fluid noradrenaline and other catecholamine metabolites (Russell et al. 1992). This is consistent with medications targeting the noradrenergic system showing some efficacy for fibromyalgia (Birkinshaw et al. 2023; Ferreira et al. 2023). Several models have been proposed to explain fibromyalgia, such as the Imbalance of Threat and Soothing Systems model (Pinto et al. 2023), which outlines possible mechanisms underlying the constellation of symptoms. In these models, the involvement of the noradrenergic system is consistent with its established role in modulating responses to threat and pain (Pertovaara 2006; Uematsu et al. 2017).

The primary source of noradrenaline in the central nervous system is the locus coeruleus (LC; Benarroch 2018; Schwarz and Luo 2015), a midbrain nucleus with projections to the brain and spinal cord. LC activity plays a critical role in nociception (Llorca‐Torralba et al. 2016; Pertovaara 2006, 2013; Suarez‐Pereira et al. 2022) and non‐pain‐related symptoms associated with fibromyalgia, including anxiety, mood, sleep, and cognition (Wolfe et al. 2010). Noradrenaline release from the LC is a key component of the descending pain pathway for healthy antinociception (Pertovaara 2013). It is suggested that in chronic pain, the noradrenergic LC signalling switches to a facilitatory role and contributes to pain centralisation (Taylor and Westlund 2017). Chronic pain rodent models evidence generalisation to other domains, including anxiety‐like, depressive‐like and cognitive domains (Alba‐Delgado et al. 2016; Barthas et al. 2015; Yalcin et al. 2014) as in fibromyalgia (Hauser et al. 2015). These behavioural symptoms arise in tandem with changes to LC firing (Alba‐Delgado et al. 2016, 2013; McCall et al. 2015) strengthening the association between noradrenergic system function, chronic pain and associated symptoms.

A novel approach to studying the noradrenergic function of the LC utilises a paramagnetic by‐product of catecholamine synthesis, neuromelanin, using magnetic resonance imaging (MRI) (Al Haddad et al. 2023; Calarco et al. 2022; Cassidy et al. 2022, 2019; Horga et al. 2021; McCall et al. 2024; Priovoulos et al. 2020; Sibahi et al. 2023; Sulzer et al. 2018; Wengler et al. 2024). Signal intensity in the LC is considered a proxy measure of neuromelanin, reflecting lifetime noradrenaline synthesis (Keren et al. 2015), with increasing signal apparent with age up until about age 60 (Liu et al. 2019; Manaye et al. 1995; Riley et al. 2023). However, there is debate about the contributors to the LC signal as measured by MR, namely neuromelanin concentration specifically versus high free‐water content within catecholaminergic cell bodies (Trujillo et al. 2024a, 2024b; Watanabe 2024; Wengler et al. 2024). We refer to ‘LC signal’ to reflect this ongoing debate and uncertainty regarding the source of this signal.

In this study, we hypothesised that fibromyalgia patients would have a greater lifetime synthesis of noradrenaline as quantified by LC signal intensity. This directionality was informed by previous work which suggests that in chronic pain signalling switches to a facilitatory role and contributes to pain centralisation (Taylor and Westlund 2017), however, we acknowledge that the complexity of the condition and data would also support a hypothesis involving decreased noradrenergic signalling, as reflected by lower noradrenaline within the CSF (Russell et al. 1992). Therefore, our tests were two‐tailed. Additionally, we investigated associations between LC signal intensity in fibromyalgia and length of illness, anxiety, mood, cognition and sleep, hypothesising a relationship between these variables given the role of noradrenergic functioning in these domains.

## 1 Methods

This study was approved by the local University of Calgary Institutional Review Board and all participants provided informed consent.

### 1.1 Participants

We recruited male and female participants with fibromyalgia aged 18–65 years from public chronic pain clinics affiliated with the University of Calgary, through the acquaintanceship method and through advertisement. Efforts were made to match healthy controls on age and sex, including through the acquaintanceship method. For participants with fibromyalgia, eligibility criteria were: a diagnosis of fibromyalgia as per the American College of Rheumatology 2016 criteria (Wolfe et al. 2016), experience of moderate impact of symptoms, as defined by a Revised Fibromyalgia Impact Questionnaire (FIQR; Bennett et al. 2009) score of ≥ 41, ongoing symptoms. Healthy control participants were eligible provided they had no unstable medical conditions (managed health conditions, for example, dyslipidemia, were permitted), no chronic pain conditions and no MRI safety exclusion criteria.

### 1.2 Clinical Measures

Questionnaires were used to quantify the core and associated symptoms of fibromyalgia. The gold‐standard Revised Fibromyalgia Impact Questionnaire (FIQR) was used to capture the function, impact and symptoms of fibromyalgia (Bennett et al. 2009). The FIQR consists of 21 questions about the last week, rated on a 0–10 scale (with 10 being the worst), for a score out of 100 (Bennett et al. 2009). There are three subscales of the FIQR, scaled differently to create the total score. There are nine items in the physical function subscale, with the score divided by 3 to create a subscale score out of 30, 2 in the overall score which are not transformed for a total subscale score of 20 and 10 items in the symptom domain subscale, which is divided by two to a total subscale score of 50. Additionally, to determine pain intensity as a standalone measure, we used item 12 of the FIQR.

The Cognitive Failures Questionnaire (CFQ; Broadbent et al. 1982) was used to quantify subjective cognitive complaints. This 25‐item questionnaire asks participants to rate the frequency of cognitive mistakes on a scale of 0 (never) to 4 (very often), and the total score is the sum of these responses, with a high score indicating higher levels of subjective cognitive difficulties (Broadbent et al. 1982).

The presence and severity of symptoms associated with central sensitivity syndromes was assessed with the Central Sensitization Inventory (CSI), Part A (Mayer et al. 2012). This contains 25 items, resulting in a score out of 100, with higher scores indicating greater endorsement of symptoms related to central sensitization syndromes (Mayer et al. 2012).

The Insomnia Severity Index (ISI) was used to quantify sleep disturbance and associated daytime difficulties (Bastien et al. 2001). The 7 items assess difficulties with initiating and maintaining sleep, sleep satisfaction, the impact of sleep on daily function and sleep‐related distress for the preceding 2 weeks. These items are rated on a 0–4 scale, with a total maximum score of 28 (Bastien et al. 2001).

To assess levels of anxiety, we used the General Anxiety Disorder—7‐item scale (GAD‐7), which queries the frequency of various anxious symptoms over the last 2 weeks (Spitzer et al. 2006).

For measurement of depressive symptoms, we used the 16‐item Quick Inventory of Depressive Symptomatology—Self Report (QIDS‐SR), which results in a total score out of 27, with higher scores indicating higher levels of depressive symptoms (Rush et al. 2003).

Participants self‐reported their symptomatic, diagnostic and medication history.

### 1.3 Cognitive Measurements

To measure cognitive function, participants completed the THINC‐it, a computerised cognitive battery (McIntyre et al. 2017). It is comprised of five sub‐tests based on established cognitive tests:

1. The ‘Spotter’ sub‐test is based on the Choice Reaction Time (CRT) involves 40 trials in which subjects are presented with an arrow facing either left or right. Participants are asked to press a button corresponding to the direction of the arrow. Latency to response is recorded, and shorter reaction time indicates better performance and attention.
2. The ‘Symbol Check’ sub‐test is based on the 1‐Back test. It involves 40 trials in which a continuously moving sequence of symbols moves across the screen. As the sequence moves to the left, the participant is asked to identify the previous symbol, now hidden, before time‐out (3 s). A legend of 5 possible symbols is presented at the bottom of the screen. Accuracy in the form of number correct is recorded, with higher number of correctly identified symbols indicating better performance and working memory.
3. The ‘Trails’ sub‐test is based on Part B of the Trail Making Test. Participants are asked to trace a line connecting consecutive numbers and letters in alternation. If the line touches a number or letter out of sequence, the participant must restart beginning at ‘A'. Completion time is recorded, with lower times indicating better performance and executive function.
4. The ‘Codebreaker’ sub‐test is based on the Digital Symbol Substitution Test (DSST). Participants are presented with a series of numbers and are asked to match numbers with its associated symbol within 2 min. The corresponding legend of 6 symbols is located at the top of the screen. The number of correct responses is recorded, with more correct symbols indicative of better performance and cognitive function across several domains including working memory, attention and executive function
5. The ‘Perceived Difficulties Questionnaire’ (PDQ) sub‐test is a five‐item questionnaire, which quantifies perceived challenges with attention, memory, and concentration in the last 7 days. Each item is rated on a Likert scale ranging from 1 (‘Never’) to 5 (‘Very Often [More than once a day]’). The total score is recorded, and higher scores are indicative of greater subjective cognitive impairment.

The THINC‐it has been validated relative to the original cognitive tests (Harrison et al. 2018), demonstrated to be sensitive to cognitive deficits (McIntyre et al. 2017), and to have acceptable test–retest reliability (Dalby et al. 2022; Harrison et al. 2018).

### 1.4 MRI Acquisition

**FIGURE 1:**
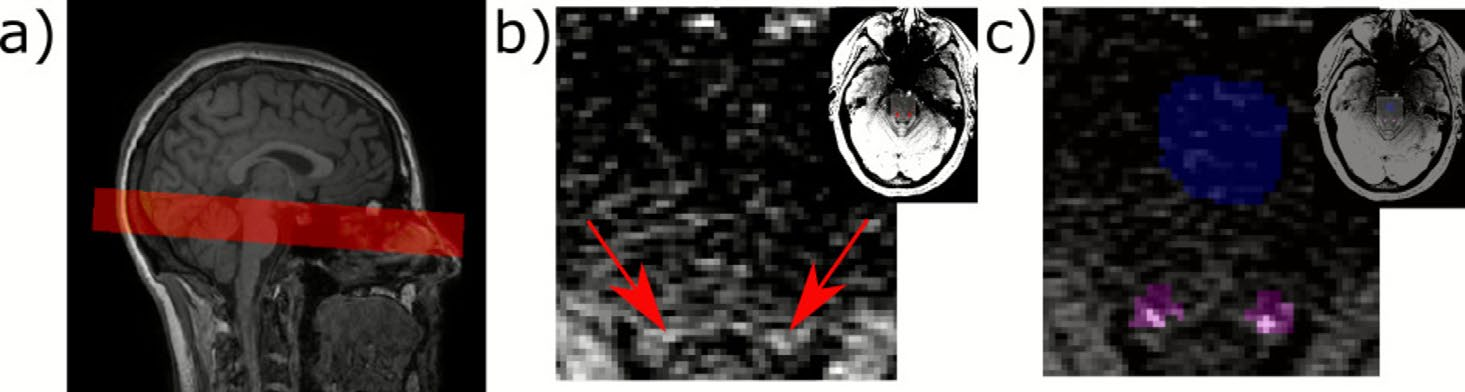
(a) example of slice stack placement for gradient echo scan for quantification of locus coeruleus (LC) signal intensity, (b) illustrative slice of the gradient scan with the LC signal highlighted by the arrows, (c) example of the regions of interest for the analysis: LC search area identified from atlas shown in pink, the 6 voxels in this slice with identified LC signal shown in white and the pons reference region in blue.

MRI data acquisition was acquired on two MRI systems due to a significant scanner upgrade. Both acquisitions included a detailed T1‐weighted anatomical scan and a 2D gradient echo sequence. The gradient echo sequence was based on previous work by Sibahi et al. (2023), with scanner‐specific optimisations for each scanner investigating flip angle, repetition time (TR) and echo time (TE), aiming to minimise cerebrospinal fluid (CSF) contamination and enhance contrast from within the LC. The T1‐weighted anatomical scan was used for placement and coregistration of the 2D gradient echo sequence. The 2D gradient echo sequence was positioned perpendicular to the brainstem, with the most inferior slice positioned in line with the posterior recess of the fourth ventricle. An example of the slice stack placement is shown in Figure 1a.

Pre‐upgrade data was acquired on a 3 T General Electric MR750 Discovery scanner, utilising a 3D spoiled gradient recall for the T1‐weighted anatomical scan (TR = 8.1 ms, TE = 3.2 ms, 1 mm isotropic voxels). The sequence for LC signal contrast was a 2D gradient echo acquisition, with the following parameters: TR = 475 ms, FA = 50 degrees, TE = 4.7 ms, 0.47 × 0.47 × 1.5 mm voxels reconstructed, 5 averages, 16 slices with a 1200 ms magnetisation transfer pulse, taking 9 min, 24 s.

Post‐upgrade, a 3 T General Electric Signa Ultra High‐Performance MR scanner was used. The T1‐weighted anatomical was a 3D BRAin Volume T1 (TR = 6.712 ms, TE = 2.748 ms, voxel size = 0.43 × 0.43 × 1 mm, flip angle = 10°, phase acceleration = 2, slice acceleration = 1.5, ARC GE deep learning strength = medium). For the measurement of LC signal contrast, a 2D gradient echo acquisition, with the parameters of TR = 525 ms, TE = 4.3 ms, FA = 60 degrees, 0.47 × 0.47 × 1.8 mm voxels reconstructed, 16 slices with a 1200 ms magnetisation transfer pulse. This acquisition was repeated as five separate scans to create five separate averages for offline averaging.

### 1.5 MRI Processing

Data was processed using a custom‐built, semi‐automated, MATLAB algorithm that has been validated against manual LC segmentation and has shown excellent reliability (Cassidy et al. 2022; Sibahi et al. 2023). Briefly, using the coregistration with ANTS toolbox (Avants et al. 2008, 2011) an overly inclusive mask of the LC region is registered from MNI space to each participant's unprocessed gradient echo image. This mask acts as a search region for a ‘funnel‐tip’ search method, which identifies the most intense cluster of five contiguous voxels within the LC search region within each axial slice identified to contain the LC mask. The LC search region is divided into five rostro‐caudal sections each spanning 3 mm in the z‐axis, that is, with the search region spanning 15 mm in the z direction. The pre‐upgrade and post‐upgrade data were processed separately due to the differing slice and voxel sizes. Each mask was visually inspected to ensure accuracy and adjusted in the presence of large artefacts, for example, if an artefact such as hyperintense CSF was mistakenly labelled as LC. The contrast‐to‐noise ratio (CNR) was calculated using the LC contrasted to a central pons reference region, a standard region chosen for its low neuromelanin signal (Cassidy et al. 2022; McCall et al. 2024; Sibahi et al. 2023). Outliers were identified based on exceeding three times the interquartile range and removed prior to harmonisation.

ComBat harmonisation was applied to harmonise the central LC signal between the two MRI systems. Harmonisation was completed following the quantification of the LC, prior to averaging within the selected segments. This harmonisation procedure, initially developed for genomics data (Johnson et al. 2007), uses empirical Bayes factors to adjust for batch effects and remove unwanted, non‐biological, variance and has been established for application in MR imaging data to account for MRI system effects (Bell et al. 2022; Fortin et al. 2018, 2017; Wengler et al. 2021; Yu et al. 2018). The three central segments of the LC from each side were used as the input features for harmonisation, to minimise possible signal contamination and partial volume effects from the head and tail of the LC. Covariates were age and diagnosis, with scanner as the batch effect. The signal from all voxels within the three segments was harmonised. After harmonisation, the CNR for the LC was calculated as an average of the most intense 2 of these voxels bilaterally on each axial slice assigned to the central 3 rostro‐caudal sections as a ratio to a central pons reference region and used for subsequent analyses. The choice of two voxels for the LC was informed by Sibahi et al. (2023), who found this size of LC for calculating CNR was a good balance between capturing sufficient LC and minimising partial voluming effects. Sibahi et al. (2023) had a lower spatial resolution than our study, and so to more closely match the same LC cross‐sectional area we selected two voxels rather than one. Additionally, we calculated CNR from the LC without harmonisation separately for the pre‐ and post‐upgrade data independently and replicated our analyses, reported in the Data S1. Throughout the text, we refer to the summary LC metric from the CNR calculation as LC signal.

### 1.6 Analysis

Statistical analysis was conducted in SPSS, version 29.0.1.1 with alpha set at ≤ 0.05 and G*Power, version 3.1.9.6, was used to calculate sensitivity.

Shapiro–Wilk was used to confirm the normality of variables, with non‐parametric tests used when the assumption of normality was not met. Independent group *t*‐tests, chi square or Wilcoxon Rank Sum tests were used to compare participants with fibromyalgia to healthy controls on demographic and clinical variables and harmonised LC signal. Normality was assessed for values used for the correlational analysis (whole sample: age, LC signal, measures of cognition; fibromyalgia participants: length of symptoms, time since diagnosis, questionnaire measures, measures of cognition). When the assumption of normality was not met, non‐parametric tests were used (either Spearman's rank order correlation or ordinal regression). The age‐LC signal was investigated using a Pearson's *r* correlation, within each group (fibromyalgia and healthy controls) and across the whole sample. To investigate potential group differences in the age‐LC signal relationship, a linear model was used to investigate group*age interaction on LC signal. Within participants with fibromyalgia, to investigate associations between LC signal and clinical characteristics of fibromyalgia (length of symptoms, time since diagnosis, symptom levels), partial correlations were conducted, accounting for age. To investigate possible associations between cognitive function and LC function, partial correlations were conducted between each of the THINC domains and the average LC signal, accounting for age, both within the participants with fibromyalgia and the whole sample.

## 2 Results

### 2.1 Participants

Data were collected from 46 patients with fibromyalgia and 41 healthy controls. Pre‐upgrade data were collected from 26 patients with fibromyalgia and 24 healthy controls, and post‐upgrade data were collected from 20 patients with fibromyalgia and 17 healthy controls. Demographic and clinical variables are shown in Table 1. There were no significant differences in age or sex between the groups. For those participants that reported symptom duration (*N* = 43), they had an average symptom duration of 11.05 years (SD = 9.13), with an average of 5.79 (SD = 7.03) years from symptom onset to diagnosis. Participants with fibromyalgia had greater FIQR, CFQ, CSI, QIDS‐SR, GAD‐7 and ISI scores, independent groups *t*‐tests reported in Table 1.

**TABLE 1:** Sample characteristics.

	Fibromyalgia	Healthy controls	Group differences
**Demographics**			
N	46	41	—
Age	45.67 (10.37)	45.98 (11.50)	*t*(85) = −013, *p* = 0.90
Sex at birth	2 M/44F	2 M/39F	χ^2^(1) = 0.01, *p* = 0.93
Gender identity (man/woman/non‐binary/not reported)	1 M/43 W/0NB/2NR	2 M/38 W/1NB	χ^2^(2) = 1.48, *p* = 0.48
**Questionnaire measures**			
FIQR^a^	61.99 (13.10)	4.23 (5.62)	W_s_ = 781.00, *p* < 0.001
CFQ^a^, ^b^	59.61 (13.58)	26.74 (15.82)	W_s_ = 843.50, *p* < 0.001
CSI^a^, ^b^	68.34 (9.57)	15.34 (10.00)	W_s_ = 742.00, *p* < 0.001
GAD‐7^a^, ^b^	8.88 (5.68)	1.68 (2.59)	W_s_ = 894.00, *p* < 0.001
QIDS‐SR^a^, ^b^	12.22 (5.85)	2.76 (2.61)	W_s_ = 875.50, *p* < 0.001
ISI^a^, ^b^, ^c^	18.24 (5.00)	5.24 (5.17)	W_s_ = 619.00, *p* < 0.001
**Cognitive measures as per THINC‐it**			
Spotter^d^	918.81 (290.39)	799.63 (241.86)	W_s_ = 508.00, *p* = 0.03
Symbol check^d^	17.43 (11.41)	20.95 (9.01)	W_s_ = 951.00, *p* = 0.09
Trails^d^, ^e^	32.51 (15.07)	26.36 (10.92)	W_s_ = 1295.00, *p* = 0.04
Codebreaker^d^	42.26 (14.53)	52.24 (17.10)	*t*(78) = −2.82, *p* = 0.006
Perceived difficulties^d^	19.26 (3.97)	9.11 (2.69)	W_s_ = 772.50, *p* < 0.001
**Current pain medications**			
NSAIDs	9	—	
Opioids	9	—	
Acetaminophen		—	
**Current psychotropic medications**			
Average and range of number of medications	1.67 (0–4)		
TCAs	7	—	
SSRIs	11	—	
SNRIs	19	—	
Benzodiazepines	5	—	
Stimulants	11	—	
Antipsychotics	4	—	
**Past noradrenergic exposure**			
SNRIs/TCAs	41	—	

Abbreviations: CFQ, Cognitive Failures Questionnaire; FIQR, Fibromyalgia Impact Questionnaire—Revised; GAD‐7, General Anxiety Disorder—7‐item; ISI, Insomnia Severity Index; NSAIDs, non‐steroidal anti‐inflammatory drugs; QIDS‐SR, Quick Inventory of Depressive Symptomatology—Self Report; SNRIs, serotonin–norepinephrine reuptake inhibitors; SSRIs, selective serotonin reuptake inhibitors; TCAs, tricyclic antidepressants.

^a^ Two healthy control participants did not complete the online questionnaires and therefore are missing from these values.

^b^ Three participants with fibromyalgia did not complete the online questionnaires and therefore are missing from these values.

^c^ Incomplete forms for 9 participants who were excluded.

^d^ Missing data for the THINC IT domains for 6 participants (3 healthy controls, 3 patients with fibromyalgia).

^e^ Two participants with fibromyalgia were extreme outliers on the Trails task and removed from reporting and analysis.

Visual quality control identified and remedied 23 instances of LC masking incorporating non‐LC regions, in 1215 LC slices (a 1.89% error rate). After visual inspection and prior to harmonisation, 10 scans were excluded due to: insufficient coverage of the LC (*N* = 4 participants with fibromyalgia), outliers in the LC quantification (greater than 3 interquartile ranges from the upper or lower quartile, *N* = 5; 1 with fibromyalgia, 4 healthy controls), and missing values in the central LC segments used for harmonisation (*N* = 1 healthy control). This resulted in a total sample of 41 participants with fibromyalgia (23 pre‐upgrade, 18 post‐upgrade) and 36 healthy controls (23 pre‐upgrade, 13 post‐upgrade) that had valid LC data.

This neuroimaging aim was embedded within a larger fibromyalgia study, which was not specifically powered to detect LC signal differences between healthy controls and participants with fibromyalgia. The achieved sample size had an 80% power to detect a moderate effect size at an alpha of 0.05.

### 2.2 LC Signal and Age

**FIGURE 2:**
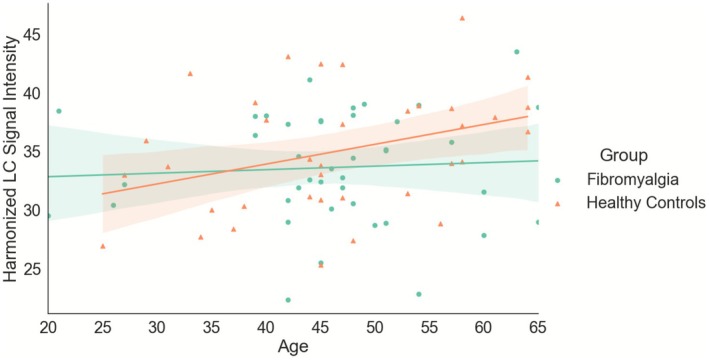
Harmonised locus coeruleus (LC) signal and age plotted for participants with fibromyalgia (green circles) and healthy controls (orange triangles).

The assumption of normality was supported for both age (W(77) = 0.97, *p* = 0.06) and LC signal (W(77) = 0.99, *p* = 0.64) and so we examined the relationship between age and LC signal. There was a significant correlation between age and LC signal in the healthy control participants (*r*(36) = 0.35, *p* = 0.04), which was not present in the participants with fibromyalgia (*r*(41) = 0.06, *p* = 0.70). Across the whole sample, there was a positive trend‐level association between age and LC signal intensity across all participants, *r*(77) = 0.21, *p* = 0.07. Given these relationships, partial correlations were used to control for age for all other correlations (Figure 2).

### 2.3 LC Signal—Group Differences

**FIGURE 3:**
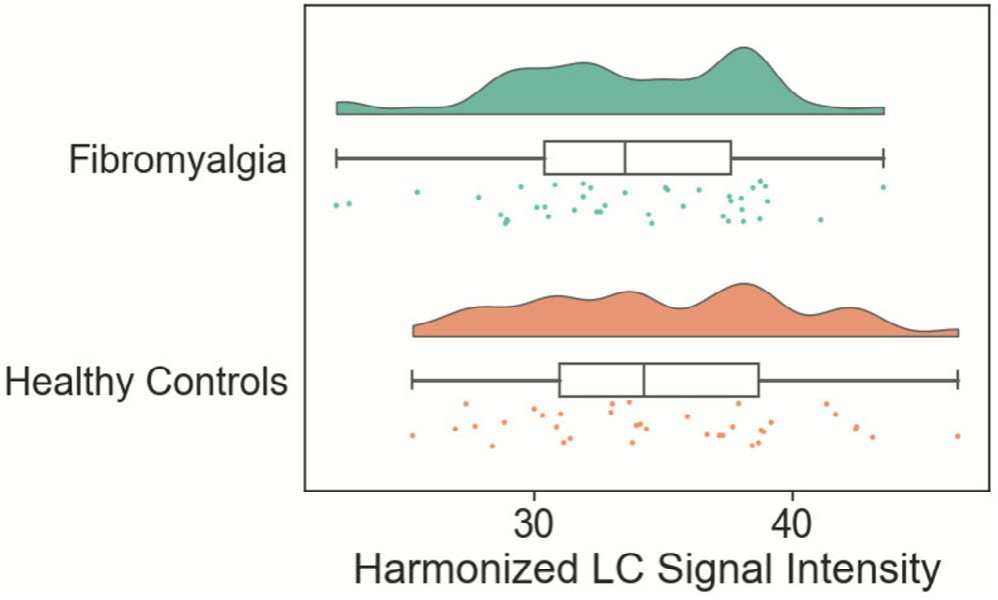
Raincloud plots showing locus coeruleus signal (LC) signal following harmonisation in the fibromyalgia (shown in green) and healthy control (shown in orange) groups. Scatterplots represent individual values from participants, with boxplots showing the interquartile range, and the whiskers extending to the most extreme value within 1.5 times the interquartile range from the hinge.

There were no group differences in the average signal of the harmonised central LC (*t*(75) = −1.19, *p* = 0.24). The average harmonised voxel contrast intensity of the LC in participants with fibromyalgia was 33.61 (SD = 4.83) and 34.97 (SD = 5.26) in healthy controls, with distributions displayed in Figure 3. To investigate potential group differences in the age‐LC signal relationship, a linear model was used to investigate group × age interaction on LC signal. This revealed a trend‐level main effect of age (*F*(1,73) = 3.36, *p* = 0.071), no significant group differences (*F*(1,73) = 0.98, *p* = 0.33) and no age × group interaction (*F*(1,73) = 1.6, *p* = 0.20). The model estimates showed a significant main effect of age (*beta* = 0.17, standard error (SE) = 0.08, *t* = 2.21, 95% confidence interval (CI) = 0.017–0.322, *p* = 0.03), but with no effect of group (fibromyalgia reference group, *beta* = 5.10, SE = 5.16, *t* = 0.99, 95% CI = −5.18–15.37, *p* = 0.33) or an interaction between group and age (fibromyalgia reference group, *beta* = −0.14, SE = 0.11, *t* = −1.28, 95% CI = −0.36 to 0.08, *p* = 0.20).

### 2.4 LC Signal—Fibromyalgia History

Given the substantial heterogeneity in LC signal, we considered whether diagnostic and symptom history influenced LC signal. The assumption of normality was met for time since symptom onset (W(37) = 0.96, *p* = 0.14) but not for time since diagnosis (W(38) = 0.93, *p* = 0.014) We found no association between time since symptom onset and LC signal intensity (*r*(35) = 0.18, *p* = 0.28) while controlling for age, nor between time since diagnosis and LC signal (Spearman's *rho*(37) = 0.03, *p* = 0.87). It was not possible to investigate the effects of medications impacting noradrenergic signalling (serotonin‐norepinephrine reuptake inhibitors (SNRIs) and secondary amine tricyclic antidepressants (TCAs)) on LC signal as only five participants with fibromyalgia did not have a history of exposure to these medications (past medications, Table 1).

### 2.5 LC Signal and Fibromyalgia Symptom Assessments

The fibromyalgia group had significantly higher scores on measures of associated symptoms (Table 1), therefore the associations with LC signal and these measures were conducted only within the participants with fibromyalgia. All of the clinical questionnaires and THINC‐it domains met the assumption of normality (all *p* > 0.05), with the exception of the Symbol‐Check working memory measure (*W* = 0.92, *p* = 0.012) and question 12 from the FIQR (*W* = 0.93, *p* = 0.014). Partial correlations accounting for age did not show any association between LC signal intensity and FIQR (*r*(41) = 0.11, *p* = 0.50), the Central Sensitization Index (*r*(37) = 0.17, *p* = 0.33), the Insomnia Severity Index (*r*(35) = 0.10, *p* = 0.58), the GAD‐7 Anxiety measure (*r*(37) = −0.9, *p* = 0.59) or the QIDS‐SR Depression Measure (*r*(36) = −0.22, *p* = 0.19). Given the limited range of the FIQR question 12 for pain intensity, it was treated as an ordinal variable and ordinal regression was used to investigate if LC signal was predictive of item 12 of the FIQR. This model showed no significant relationship (chi(1) = 0.003. *p* = 0.96).

Investigating the association of cognition and LC signal, there was no relationship between LC signal and the CFQ (*r*(37) = 0.09, *p* = 0.62) among fibromyalgia participants, controlling for age. When investigating cognitive performance as measured by the THINC‐it, controlling for age, there were no associations between LC signal and the ‘Spotter’ measure of attention (*r*(32) = 0.32, *p* = 0.06), the ‘Trails’ measure of executive function (*r*(34) = −0.07, *p* = 0.68), the ‘Codebreaker’ measure of multiple cognitive domains (*r*(35) = −0.05, *p* = 0.77), the ‘Symbol Check’ working memory measure (Spearman's *rho*(37) = −0.03, *p* = 0.84) or the ‘Perceived Difficulties Questionnaire’ (PDQ) assessment of subjective cognitive function (*r*(34) = 0.03, *p* = 0.84).

Within the healthy control sample, investigating the association LC signal and cognitive performance as measured by the THINC‐it, controlling for age, there were no associations between LC signal and the ‘Spotter’ measure of attention (*r*(30) = 0.07, *p* = 0.72), the ‘Symbol Check’ working memory measure (*r*(30) = −0.05, *p* = 0.81), the ‘Trails’ measure of executive function (*r*(30) = −0.12, *p* = 0.52), the ‘Codebreaker’ measure of multiple cognitive domains (*r*(30) = 0.08, *p* = 0.86) or the ‘Perceived Difficulties Questionnaire’ (PDQ) assessment of subjective cognitive function (*r*(30) = 0.07, *p* = 0.72).

Unlike other associated symptoms, cognitive performance as measured by the THINC‐it was not bimodal allowing us to test the association between LC signal and cognition in the pooled sample, and therefore greater statistical power. While controlling for age, investigation of average LC signal intensity with each of the THINC‐it measures across the whole sample revealed no significant associations in the ‘Spotter’ measure of attention (*r*(64) = 0.17, *p* = 0.18), the ‘Symbol Check’ working memory measure (*r*(67) = −0.08, *p* = 0.50), the ‘Trails’ measure of executive function (*r*(67) = −0.03, *p* = 0.81), the ‘Codebreaker’ measure of multiple cognitive domains (*r*(67) = 0.05, *p* = 0.70) or the ‘Perceived Difficulties Questionnaire’ (PDQ) assessment of subjective cognitive function (*r*(67) = −0.09, *p* = 0.46).

## 3 Discussion

Although LC signal alterations have been reported in chronic pain (Bell et al. 2021), studies specifically examining fibromyalgia are limited. Contrary to our hypotheses, we found no group differences in LC signal between participants with fibromyalgia and healthy controls, and LC signal was not associated with fibromyalgia natural history. A priori, we also hypothesised that LC signal would be associated with non‐pain symptom domains implicated in fibromyalgia, such as anxiety, mood, sleep and cognition; yet similarly did not find an association between LC signal and these associated symptoms. As an internal control for the validity of our ascertainment of LC signal, we replicated the established association between LC signal and age in our healthy control participants, with increasing LC signal with increasing age, consistent with the literature that shows increasing LC signal until approximately age 60 (Liu et al. 2019; Manaye et al. 1995; Riley et al. 2023). It is important to note that this relationship was not observed among individuals with fibromyalgia, potentially reflecting heterogeneity in the population and noradrenergic function across the lifespan.

The confirmation of a relationship between age and LC signal (Liu et al. 2019; Manaye et al. 1995; Mann et al. 1985; Riley et al. 2023) in our healthy control sample is an important internal control and validation of the method, lending credence to the null group difference findings and lack of association with symptom domains. Previous work has shown accumulation with age in the age range of this study (Liu et al. 2019; Manaye et al. 1995; Mann et al. 1985). In the older adult, however, a different relationship emerges (Liu et al. 2019; Manaye et al. 1995; Mann et al. 1985). Previous work suggests that LC signal increases until approximately age 60, at which point LC signal appears to begin to decrease (Liu et al. 2019, 2020). This decrease has been interpreted as a decline of the integrity of the nucleus (Betts et al. 2019; Cassidy et al. 2022; Engels‐Dominguez et al. 2024; Guinea‐Izquierdo et al. 2021; Ye et al. 2023). Age‐related differences may explain or contribute to our null finding regarding LC signal and cognitive performance. Previous studies that have shown relationships between LC signal and cognition were conducted in older adults (Clewett et al. 2016; Liu et al. 2020; Ye et al. 2023). These studies may be capturing the decline of the nucleus, not yet present in this sample.

When studying fibromyalgia, it is critical to consider that it is a complex condition; while characterised by chronic and diffuse somatic symptoms, it is also associated with ‘flares’ during which pain symptoms are distinctly more functionally impairing (Vincent et al. 2016). Our hypothesis of a higher LC signal in fibromyalgia was derived from LC signal being a lifetime marker of catecholamine synthesis in the LC and therefore reflects both state and trait fluctuations in pathology. A cross‐sectional marker of lifetime synthesis may not be sensitive to the pathological processes involved in fibromyalgia. Alternatively, fibromyalgia may be a heterogeneous condition with multiple pathways to a common phenotype. Moreover, although the role of the LC in modulating pain and associated affective, behavioural and cognitive sequelae is commonly studied in models of chronic pain (Alba‐Delgado et al. 2016; Barthas et al. 2015; Yalcin et al. 2014), there is no model for fibromyalgia to inform or test mechanisms of disease. Accordingly, findings from chronic pain may not extend to fibromyalgia, consistent with the overall challenge in identifying pharmacological agents that are effective in fibromyalgia treatment (Alorfi 2022; Hauser et al. 2014, 2012).

Alternatively, the high prevalence of comorbid conditions with fibromyalgia that also implicate noradrenergic signalling may reduce signal to noise and specificity of the LC signal. Previous LC signal MRI studies have shown reduced LC signal in depression (Guinea‐Izquierdo et al. 2021; Shibata et al. 2007), while the opposite appears to be true with anxiety (Morris et al. 2020) (McCall et al. 2024). The opposite direction of effect for these two common comorbidities in fibromyalgia may conflate the LC signal and prevent the identification of a ‘pure’ fibromyalgia effect. Although we attempted to address this by examining relationships between current symptoms and LC signal, this approach does not capture the natural history of the comorbidities and their effects on LC signal. Moreover, this study was not powered to investigate effects of medications or comorbidities on LC signal, a valuable direction for future research in this population.

### 3.1 Limitations

We acknowledge that, particularly in older adults, LC signal may alternatively reflect the structural integrity of the nucleus (Betts et al. 2019; Calarco et al. 2022; Cassidy et al. 2022; Engels‐Dominguez et al. 2024; Guinea‐Izquierdo et al. 2021; Liu et al. 2019, 2020; Ye et al. 2023). Indeed, lower rostral LC integrity has been reported in males over 65 with chronic pain (Bell et al. 2021). Given the age range of participants in the present study, LC signal intensity is more likely to reflect lifespan LC function rather than LC nucleus integrity; however, alterations in LC integrity cannot be entirely ruled out.

This study considered a wide range of ages. Future investigations may consider focusing on the older adult population where changes to LC signal might become more apparent. Alternatively, focussing on a younger adult population may limit the heterogeneity of LC signal intensity across the lifespan. There would be value in a longitudinal rather than cross‐sectional design, especially for capturing the impact of symptoms on LC signal. Similarly, future research would benefit from deeper fibromyalgia phenotyping to explore the impacts of comorbid conditions and medication history. It is also worth noting that our cognitive measures were a brief screening cognitive measure, and that more in‐depth testing may have greater sensitivity to noradrenergic dysfunction.

There are several inherent limitations to the LC signal measure. Notably, it provides an indirect measure of lifetime noradrenergic synthesis, does not reflect dynamic changes and has additional contributions of free‐water content to the signal (Trujillo et al. 2024a, 2024b; Watanabe 2024; Wengler et al. 2024). LC signal has also not been validated as a marker of noradrenergic function with PET in healthy, non‐neurodegenerative populations (Wengler et al. 2024), and previous validation studies in healthy tissue focused on dopamine and neuromelanin in the substantia nigra (Cassidy et al. 2019). Finally, it is worth noting that the data collection for this study crossed a scanner upgrade and the two acquisitions differed in several parameters, including TR, TE and voxel size. As a result, the two sequences differed in signal intensity, an issue addressed through data harmonisation.

## 4 Conclusion

This study investigated LC signal intensity in participants with fibromyalgia and healthy controls. Our analyses do not support the hypothesis that lifetime catecholamine synthesis in the LC, as indexed by LC signal intensity, is associated with fibromyalgia diagnosis, its pain and non‐pain symptom domains. Special consideration of age effects and heterogeneity in the syndrome will be required in future research.

## Author Contributions

This study was designed by M.M.D., A.D.H, and A.M. T.T., A.D.H, and A.M. procured funding and ethical approval for the study. A.D.H, and A.M. supervised the research project. M.M.D., C.M.C., C.M. and A.D.H. implemented and optimised the acquisition. C.M.C. provided the initial acquisition and full analysis pipeline. The data were analysed by M.M.D., A.D.H, and A.M., and the results were critically examined by all authors. M.M.D. had a primary role in preparing the manuscript, which was edited by all authors. All authors have approved the final version of the manuscript and agree to be accountable for all aspects of the work.

## Supporting information


**Data S1:** Supporting information.

## Data Availability

Data will be made available upon reasonable request to the corresponding author.

## References

[ejp70173-bib-0001] Al Haddad, R. , M. Chamoun , C. L. Tardif , et al. 2023. “Normative Values of Neuromelanin‐Sensitive MRI Signal in Older Adults Obtained Using a Turbo Spin Echo Sequence.” Journal of Magnetic Resonance Imaging 58, no. 1: 294–300. 10.1002/jmri.28530. 36373996

[ejp70173-bib-0002] Alba‐Delgado, C. , A. Cebada‐Aleu , J. A. Mico , and E. Berrocoso . 2016. “Comorbid Anxiety‐Like Behavior and Locus Coeruleus Impairment in Diabetic Peripheral Neuropathy: A Comparative Study With the Chronic Constriction Injury Model.” Progress in Neuro‐Psychopharmacology & Biological Psychiatry 71: 45–56. 10.1016/j.pnpbp.2016.06.007. 27328428

[ejp70173-bib-0003] Alba‐Delgado, C. , M. Llorca‐Torralba , I. Horrillo , et al. 2013. “Chronic Pain Leads to Concomitant Noradrenergic Impairment and Mood Disorders.” Biological Psychiatry 73, no. 1: 54–62. 10.1016/j.biopsych.2012.06.033. 22854119

[ejp70173-bib-0004] Alorfi, N. M. 2022. “Pharmacological Treatments of Fibromyalgia in Adults; Overview of Phase IV Clinical Trials.” Frontiers in Pharmacology 13: 1017129. 10.3389/fphar.2022.1017129. 36210856 PMC9537626

[ejp70173-bib-0005] Avants, B. B. , C. L. Epstein , M. Grossman , and J. C. Gee . 2008. “Symmetric Diffeomorphic Image Registration With Cross‐Correlation: Evaluating Automated Labeling of Elderly and Neurodegenerative Brain.” Medical Image Analysis 12, no. 1: 26–41. 10.1016/j.media.2007.06.004. 17659998 PMC2276735

[ejp70173-bib-0006] Avants, B. B. , N. J. Tustison , G. Song , P. A. Cook , A. Klein , and J. C. Gee . 2011. “A Reproducible Evaluation of ANTs Similarity Metric Performance in Brain Image Registration.” NeuroImage 54, no. 3: 2033–2044. 10.1016/j.neuroimage.2010.09.025. 20851191 PMC3065962

[ejp70173-bib-0007] Barthas, F. , J. Sellmeijer , S. Hugel , E. Waltisperger , M. Barrot , and I. Yalcin . 2015. “The Anterior Cingulate Cortex Is a Critical Hub for Pain‐Induced Depression.” Biological Psychiatry 77, no. 3: 236–245. 10.1016/j.biopsych.2014.08.004. 25433903

[ejp70173-bib-0008] Bastien, C. H. , A. Vallieres , and C. M. Morin . 2001. “Validation of the Insomnia Severity Index as an Outcome Measure for Insomnia Research.” Sleep Medicine 2, no. 4: 297–307. 10.1016/s1389-9457(00)00065-4. 11438246

[ejp70173-bib-0009] Bell, T. K. , K. J. Godfrey , A. L. Ware , K. O. Yeates , and A. D. Harris . 2022. “Harmonization of Multi‐Site MRS Data With ComBat.” NeuroImage 257: 119330. 10.1016/j.neuroimage.2022.119330. 35618196

[ejp70173-bib-0010] Bell, T. R. , C. E. Franz , L. T. Eyler , et al. 2021. “Locus Coeruleus Integrity in Older Adults With and Without Chronic Pain.” medRxiv, 2021.2011.2002.21265820.

[ejp70173-bib-0011] Benarroch, E. E. 2018. “Locus Coeruleus.” Cell and Tissue Research 373, no. 1: 221–232. 10.1007/s00441-017-2649-1. 28687925

[ejp70173-bib-0012] Bennett, R. M. , R. Friend , K. D. Jones , R. Ward , B. K. Han , and R. L. Ross . 2009. “The Revised Fibromyalgia Impact Questionnaire (FIQR): Validation and Psychometric Properties.” Arthritis Research & Therapy 11, no. 4: R120. 10.1186/ar2783. 19664287 PMC2745803

[ejp70173-bib-0013] Betts, M. J. , E. Kirilina , M. C. G. Otaduy , et al. 2019. “Locus Coeruleus Imaging as a Biomarker for Noradrenergic Dysfunction in Neurodegenerative Diseases.” Brain 142, no. 9: 2558–2571. 10.1093/brain/awz193. 31327002 PMC6736046

[ejp70173-bib-0014] Birkinshaw, H. , C. M. Friedrich , P. Cole , et al. 2023. “Antidepressants for Pain Management in Adults With Chronic Pain: A Network Meta‐Analysis.” Cochrane Database of Systematic Reviews 5, no. 5: CD014682. 37160297 10.1002/14651858.CD014682.pub2 PMC10169288

[ejp70173-bib-0015] Broadbent, D. E. , P. F. Cooper , P. FitzGerald , and K. R. Parkes . 1982. “The Cognitive Failures Questionnaire (CFQ) and Its Correlates.” British Journal of Clinical Psychology 21, no. 1: 1–16. 10.1111/j.2044-8260.1982.tb01421.x. 7126941

[ejp70173-bib-0016] Calarco, N. , C. M. Cassidy , B. Selby , et al. 2022. “Associations Between Locus Coeruleus Integrity and Diagnosis, Age, and Cognitive Performance in Older Adults With and Without Late‐Life Depression: An Exploratory Study.” Neuroimage Clinical 36: 103182. 10.1016/j.nicl.2022.103182. 36088841 PMC9474922

[ejp70173-bib-0017] Cassidy, C. M. , J. Therriault , T. A. Pascoal , et al. 2022. “Association of Locus Coeruleus Integrity With Braak Stage and Neuropsychiatric Symptom Severity in Alzheimer's Disease.” Neuropsychopharmacology 47, no. 5: 1128–1136. 10.1038/s41386-022-01293-6. 35177805 PMC8938499

[ejp70173-bib-0018] Cassidy, C. M. , F. A. Zucca , R. R. Girgis , et al. 2019. “Neuromelanin‐Sensitive MRI as a Noninvasive Proxy Measure of Dopamine Function in the Human Brain.” Proceedings of the National Academy of Sciences of the United States of America 116, no. 11: 5108–5117. 10.1073/pnas.1807983116. 30796187 PMC6421437

[ejp70173-bib-0019] Clauw, D. J. 2009. “Fibromyalgia: An Overview.” American Journal of Medicine 122, no. 12: S3–S13. 10.1016/j.amjmed.2009.09.006. 19962494

[ejp70173-bib-0020] Clewett, D. V. , T. H. Lee , S. Greening , A. Ponzio , E. Margalit , and M. Mather . 2016. “Neuromelanin Marks the Spot: Identifying a Locus Coeruleus Biomarker of Cognitive Reserve in Healthy Aging.” Neurobiology of Aging 37: 117–126. 10.1016/j.neurobiolaging.2015.09.019. 26521135 PMC5134892

[ejp70173-bib-0021] Dalby, M. , P. Annas , T. Me Research , and J. E. Harrison . 2022. “Further Validation of the THINC‐It Tool and Extension of the Normative Data Set in a Study of *n* = 10.019 Typical Controls.” International Journal of Methods in Psychiatric Research 31, no. 4: e1922. 10.1002/mpr.1922. 35748111 PMC9720188

[ejp70173-bib-0022] Engels‐Dominguez, N. , J. M. Riphagen , M. Van Egroo , et al. 2024. “Lower Locus Coeruleus Integrity Signals Elevated Entorhinal Tau and Clinical Progression in Asymptomatic Older Individuals.” Annals of Neurology 96, no. 4: 650–661. 10.1002/ana.27022. 39007398 PMC11534559

[ejp70173-bib-0023] Ferreira, G. E. , C. Abdel‐Shaheed , M. Underwood , et al. 2023. “Efficacy, Safety, and Tolerability of Antidepressants for Pain in Adults: Overview of Systematic Reviews.” BMJ 380: e072415. 10.1136/bmj-2022-072415. 36725015 PMC9887507

[ejp70173-bib-0024] Fortin, J.‐P. , N. Cullen , Y. I. Sheline , et al. 2018. “Harmonization of Cortical Thickness Measurements Across Scanners and Sites.” NeuroImage 167: 104–120. 10.1016/j.neuroimage.2017.11.024. 29155184 PMC5845848

[ejp70173-bib-0025] Fortin, J.‐P. , D. Parker , B. Tunç , et al. 2017. “Harmonization of Multi‐Site Diffusion Tensor Imaging Data.” NeuroImage 161: 149–170. 10.1016/j.neuroimage.2017.08.047. 28826946 PMC5736019

[ejp70173-bib-0026] Guinea‐Izquierdo, A. , M. Gimenez , I. Martinez‐Zalacain , et al. 2021. “Lower Locus Coeruleus MRI Intensity in Patients With Late‐Life Major Depression.” PeerJ 9: e10828. 10.7717/peerj.10828. 33628639 PMC7894108

[ejp70173-bib-0027] Harrison, J. E. , H. Barry , B. T. Baune , et al. 2018. “Stability, Reliability, and Validity of the THINC‐It Screening Tool for Cognitive Impairment in Depression: A Psychometric Exploration in Healthy Volunteers.” International Journal of Methods in Psychiatric Research 27, no. 3: e1736. 10.1002/mpr.1736. 30088298 PMC6174931

[ejp70173-bib-0028] Hauser, W. , J. Ablin , M. A. Fitzcharles , et al. 2015. “Fibromyalgia.” Nature Reviews Disease Primers 1: 15022. 10.1038/nrdp.2015.22. 27189527

[ejp70173-bib-0029] Hauser, W. , B. Walitt , M. A. Fitzcharles , and C. Sommer . 2014. “Review of Pharmacological Therapies in Fibromyalgia Syndrome.” Arthritis Research & Therapy 16, no. 1: 201. 10.1186/ar4441. 24433463 PMC3979124

[ejp70173-bib-0030] Hauser, W. , F. Wolfe , T. Tolle , N. Uceyler , and C. Sommer . 2012. “The Role of Antidepressants in the Management of Fibromyalgia Syndrome: A Systematic Review and Meta‐Analysis.” CNS Drugs 26, no. 4: 297–307. 10.2165/11598970-000000000-00000. 22452526

[ejp70173-bib-0031] Horga, G. , K. Wengler , and C. M. Cassidy . 2021. “Neuromelanin‐Sensitive Magnetic Resonance Imaging as a Proxy Marker for Catecholamine Function in Psychiatry.” JAMA Psychiatry 78, no. 7: 788–789. 10.1001/jamapsychiatry.2021.0927. 34009285 PMC9060608

[ejp70173-bib-0032] Johnson, W. E. , C. Li , and A. Rabinovic . 2007. “Adjusting Batch Effects in Microarray Expression Data Using Empirical Bayes Methods.” Biostatistics 8, no. 1: 118–127. 10.1093/biostatistics/kxj037. 16632515

[ejp70173-bib-0033] Keren, N. I. , S. Taheri , E. M. Vazey , et al. 2015. “Histologic Validation of Locus Coeruleus MRI Contrast in Post‐Mortem Tissue.” NeuroImage 113: 235–245. 10.1016/j.neuroimage.2015.03.020. 25791783 PMC4649944

[ejp70173-bib-0034] Liu, K. Y. , J. Acosta‐Cabronero , A. Cardenas‐Blanco , et al. 2019. “In Vivo Visualization of Age‐Related Differences in the Locus Coeruleus.” Neurobiology of Aging 74: 101–111. 10.1016/j.neurobiolaging.2018.10.014. 30447418 PMC6338679

[ejp70173-bib-0035] Liu, K. Y. , R. A. Kievit , K. A. Tsvetanov , et al. 2020. “Noradrenergic‐Dependent Functions Are Associated With Age‐Related Locus Coeruleus Signal Intensity Differences.” Nature Communications 11, no. 1: 1712. 10.1038/s41467-020-15410-w. PMC7136271 32249849

[ejp70173-bib-0036] Llorca‐Torralba, M. , G. Borges , F. Neto , J. A. Mico , and E. Berrocoso . 2016. “Noradrenergic Locus Coeruleus Pathways in Pain Modulation.” Neuroscience 338: 93–113. 10.1016/j.neuroscience.2016.05.057. 27267247

[ejp70173-bib-0037] Manaye, K. F. , D. D. McIntire , D. M. Mann , and D. C. German . 1995. “Locus Coeruleus Cell Loss in the Aging Human Brain: A Non‐Random Process.” Journal of Comparative Neurology 358, no. 1: 79–87. 10.1002/cne.903580105. 7560278

[ejp70173-bib-0038] Mann, D. M. , P. O. Yates , and B. Marcyniuk . 1985. “Correlation Between Senile Plaque and Neurofibrillary Tangle Counts in Cerebral Cortex and Neuronal Counts in Cortex and Subcortical Structures in Alzheimer's Disease.” Neuroscience Letters 56, no. 1: 51–55. 10.1016/0304-3940(85)90439-2. 4011048

[ejp70173-bib-0039] Mayer, T. G. , R. Neblett , H. Cohen , et al. 2012. “The Development and Psychometric Validation of the Central Sensitization Inventory.” Pain Practice 12, no. 4: 276–285. 10.1111/j.1533-2500.2011.00493.x. 21951710 PMC3248986

[ejp70173-bib-0040] McCall, A. , R. Forouhandehpour , S. Celebi , et al. 2024. “Evidence for Locus Coeruleus‐Norepinephrine System Abnormality in Military Posttraumatic Stress Disorder Revealed by Neuromelanin‐Sensitive Magnetic Resonance Imaging.” Biological Psychiatry 96, no. 4: 268–277. 10.1016/j.biopsych.2024.01.013. 38296219

[ejp70173-bib-0041] McCall, J. G. , R. Al‐Hasani , E. R. Siuda , et al. 2015. “CRH Engagement of the Locus Coeruleus Noradrenergic System Mediates Stress‐Induced Anxiety.” Neuron 87, no. 3: 605–620. 10.1016/j.neuron.2015.07.002. 26212712 PMC4529361

[ejp70173-bib-0042] McIntyre, R. S. , M. W. Best , C. R. Bowie , et al. 2017. “The THINC‐Integrated Tool (THINC‐It) Screening Assessment for Cognitive Dysfunction: Validation in Patients With Major Depressive Disorder.” Journal of Clinical Psychiatry 78, no. 7: 873–881. 10.4088/JCP.16m11329. 28858441

[ejp70173-bib-0043] McNally, J. D. , D. A. Matheson , and V. S. Bakowsky . 2006. “The Epidemiology of Self‐Reported Fibromyalgia in Canada.” Chronic Diseases in Canada 27, no. 1: 9–16. 16672135

[ejp70173-bib-0044] Morris, L. S. , A. Tan , D. A. Smith , et al. 2020. “Sub‐Millimeter Variation in Human Locus Coeruleus Is Associated With Dimensional Measures of Psychopathology: An In Vivo Ultra‐High Field 7‐Tesla MRI Study.” Neuroimage Clinical 25: 102148. 10.1016/j.nicl.2019.102148. 32097890 PMC7037543

[ejp70173-bib-0045] Pertovaara, A. 2006. “Noradrenergic Pain Modulation.” Progress in Neurobiology 80, no. 2: 53–83. 10.1016/j.pneurobio.2006.08.001. 17030082

[ejp70173-bib-0046] Pertovaara, A. 2013. “The Noradrenergic Pain Regulation System: A Potential Target for Pain Therapy.” European Journal of Pharmacology 716, no. 1–3: 2–7. 10.1016/j.ejphar.2013.01.067. 23500194

[ejp70173-bib-0047] Pinto, A. M. , R. Geenen , T. D. Wager , et al. 2023. “Emotion Regulation and the Salience Network: A Hypothetical Integrative Model of Fibromyalgia.” Nature Reviews Rheumatology 19, no. 1: 44–60. 10.1038/s41584-022-00873-6. 36471023

[ejp70173-bib-0048] Priovoulos, N. , S. C. J. van Boxel , H. I. L. Jacobs , et al. 2020. “Unraveling the Contributions to the Neuromelanin‐MRI Contrast.” Brain Structure & Function 225, no. 9: 2757–2774. 10.1007/s00429-020-02153-z. 33090274 PMC7674382

[ejp70173-bib-0049] Riley, E. , N. Cicero , K. Swallow , E. De Rosa , and A. Anderson . 2023. “Locus Coeruleus Neuromelanin Accumulation and Dissipation Across the Lifespan.” BioRxiv. 10.1101/2023.10.17.562814.

[ejp70173-bib-0050] Rush, A. J. , M. H. Trivedi , H. M. Ibrahim , et al. 2003. “The 16‐Item Quick Inventory of Depressive Symptomatology (QIDS), clinician Rating (QIDS‐C), and Self‐Report (QIDS‐SR): A Psychometric Evaluation in Patients With Chronic Major Depression.” Biological Psychiatry 54, no. 5: 573–583. 10.1016/s0006-3223(02)01866-8. 12946886

[ejp70173-bib-0051] Russell, I. J. , H. Vaeroy , M. Javors , and F. Nyberg . 1992. “Cerebrospinal Fluid Biogenic Amine Metabolites in Fibromyalgia/Fibrositis Syndrome and Rheumatoid Arthritis.” Arthritis and Rheumatism 35, no. 5: 550–556. 10.1002/art.1780350509. 1374252

[ejp70173-bib-0052] Schwarz, L. A. , and L. Luo . 2015. “Organization of the Locus Coeruleus‐Norepinephrine System.” Current Biology 25, no. 21: R1051–R1056. 10.1016/j.cub.2015.09.039. 26528750

[ejp70173-bib-0053] Shibata, E. , M. Sasaki , K. Tohyama , K. Otsuka , and A. Sakai . 2007. “Reduced Signal of Locus Ceruleus in Depression in Quantitative Neuromelanin Magnetic Resonance Imaging.” Neuroreport 18, no. 5: 415–418. 10.1097/WNR.0b013e328058674a. 17496795

[ejp70173-bib-0054] Sibahi, A. , R. Gandhi , R. Al‐Haddad , et al. 2023. “Characterization of an Automated Method to Segment the Human Locus Coeruleus.” Human Brain Mapping 44, no. 9: 3913–3925. 10.1002/hbm.26324. 37126580 PMC10203805

[ejp70173-bib-0055] Sluka, K. A. , and D. J. Clauw . 2016. “Neurobiology of Fibromyalgia and Chronic Widespread Pain.” Neuroscience 338: 114–129. 10.1016/j.neuroscience.2016.06.006. 27291641 PMC5083139

[ejp70173-bib-0056] Spitzer, R. L. , K. Kroenke , J. B. Williams , and B. Lowe . 2006. “A Brief Measure for Assessing Generalized Anxiety Disorder: The GAD‐7.” Archives of Internal Medicine 166, no. 10: 1092–1097. 10.1001/archinte.166.10.1092. 16717171

[ejp70173-bib-0057] Suarez‐Pereira, I. , M. Llorca‐Torralba , L. Bravo , C. Camarena‐Delgado , C. Soriano‐Mas , and E. Berrocoso . 2022. “The Role of the Locus Coeruleus in Pain and Associated Stress‐Related Disorders.” Biological Psychiatry 91, no. 9: 786–797. 10.1016/j.biopsych.2021.11.023. 35164940

[ejp70173-bib-0058] Sulzer, D. , C. Cassidy , G. Horga , et al. 2018. “Neuromelanin Detection by Magnetic Resonance Imaging (MRI) and Its Promise as a Biomarker for Parkinson's Disease.” NPJ Parkinsons Disease 4: 11. 10.1038/s41531-018-0047-3. PMC5893576 29644335

[ejp70173-bib-0059] Taylor, B. K. , and K. N. Westlund . 2017. “The Noradrenergic Locus Coeruleus as a Chronic Pain Generator.” Journal of Neuroscience Research 95, no. 6: 1336–1346. 10.1002/jnr.23956. 27685982 PMC5374049

[ejp70173-bib-0060] Trujillo, P. , M. A. Aumann , and D. O. Claassen . 2024a. “Neuromelanin‐Sensitive MRI as a Promising Biomarker of Catecholamine Function.” Brain 147, no. 2: 337–351. 10.1093/brain/awad300. 37669320 PMC10834262

[ejp70173-bib-0061] Trujillo, P. , M. A. Aumann , and D. O. Claassen . 2024b. “Reply: Neuromelanin? MRI of Catecholaminergic Neurons.” Brain 147, no. 3: e27–e28. 10.1093/brain/awad394. 37979197

[ejp70173-bib-0062] Uematsu, A. , B. Z. Tan , E. A. Ycu , et al. 2017. “Modular Organization of the Brainstem Noradrenaline System Coordinates Opposing Learning States.” Nature Neuroscience 20, no. 11: 1602–1611. 10.1038/nn.4642. 28920933

[ejp70173-bib-0063] Vincent, A. , M. O. Whipple , and L. M. Rhudy . 2016. “Fibromyalgia Flares: A Qualitative Analysis.” Pain Medicine 17, no. 3: 463–468. 25586303 10.1111/pme.12676

[ejp70173-bib-0064] Watanabe, T. 2024. “Neuromelanin? MRI of Catecholaminergic Neurons.” Brain 147, no. 3: e24–e26. 10.1093/brain/awad393. 37979198

[ejp70173-bib-0065] Wengler, K. , C. Cassidy , M. van der Pluijm , et al. 2021. “Cross‐Scanner Harmonization of Neuromelanin‐Sensitive MRI for Multisite Studies.” Journal of Magnetic Resonance Imaging 54, no. 4: 1189–1199. 10.1002/jmri.27679. 33960063 PMC9036665

[ejp70173-bib-0066] Wengler, K. , P. Trujillo , C. M. Cassidy , and G. Horga . 2024. “Neuromelanin‐Sensitive MRI for Mechanistic Research and Biomarker Development in Psychiatry.” Neuropsychopharmacology 50, no. 1: 137–152. 10.1038/s41386-024-01934-y. 39160355 PMC11526017

[ejp70173-bib-0068] Wolfe, F. , D. J. Clauw , M. A. Fitzcharles , et al. 2010. “The American College of Rheumatology Preliminary Diagnostic Criteria for Fibromyalgia and Measurement of Symptom Severity.” Arthritis Care & Research (Hoboken) 62, no. 5: 600–610. 10.1002/acr.20140. 20461783

[ejp70173-bib-0067] Wolfe, F. , D. J. Clauw , M. A. Fitzcharles , et al. 2016. “2016 Revisions to the 2010/2011 Fibromyalgia Diagnostic Criteria.” Seminars in Arthritis and Rheumatism 46, no. 3: 319–329. 10.1016/j.semarthrit.2016.08.012. 27916278

[ejp70173-bib-0069] Yalcin, I. , F. Barthas , and M. Barrot . 2014. “Emotional Consequences of Neuropathic Pain: Insight From Preclinical Studies.” Neuroscience and Biobehavioral Reviews 47: 154–164. 10.1016/j.neubiorev.2014.08.002. 25148733

[ejp70173-bib-0070] Ye, R. , F. H. Hezemans , C. O'Callaghan , et al. 2023. “Locus Coeruleus Integrity Is Linked to Response Inhibition Deficits in Parkinson's Disease and Progressive Supranuclear Palsy.” Journal of Neuroscience 43, no. 42: 7028–7040. 10.1523/JNEUROSCI.0289-22.2023. 37669861 PMC10586538

[ejp70173-bib-0071] Yu, M. , K. A. Linn , P. A. Cook , et al. 2018. “Statistical Harmonization Corrects Site Effects in Functional Connectivity Measurements From Multi‐Site fMRI Data.” Human Brain Mapping 39, no. 11: 4213–4227. 10.1002/hbm.24241. 29962049 PMC6179920

