# Supplementary material for: Locus Coeruleus MR Measured Signal Intensity in Fibromyalgia Relative to Healthy Controls
Source: Eur J Pain. 2025 Nov 17;29(10):e70173. doi: 10.1002/ejp.70173 (PMC12621157; doi:10.1002/ejp.70173)
Supplement: Supplementary file 1 — Data S1: Supporting information. [file EJP-29-0-s001.docx]

# Supplementary Information

# Harmonization

LC signal was calculated from the most intense bilateral two voxels prior to COMBAT harmonization and exhibited a significant difference in signal intensity between the acquisitions, *t*(71.76) = 11.26, *p* < 0.001, and shown in Supplementary Figure 1.


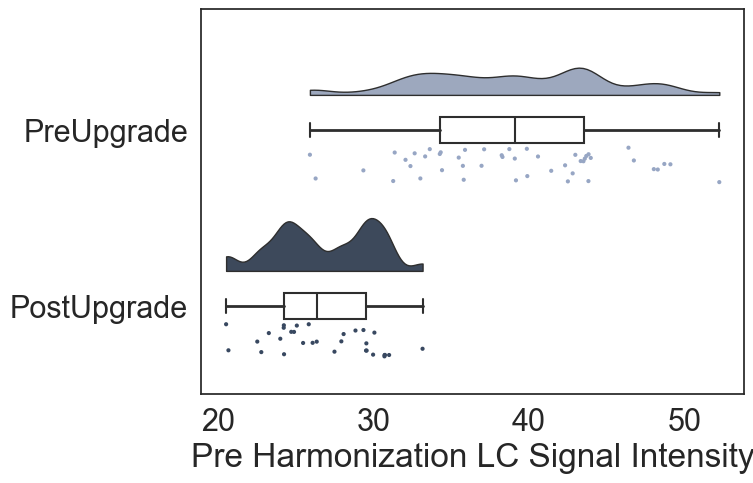


*Supplementary Figure 1. Pre-Harmonization LC Signal Intensity values by scanner status (Pre-Upgrade: 3T General Electric MR750 Discovery scanner, Post-Upgrade: a 3T General Electric Signa Ultra High-Performance MR scanner)*

Subsequent to harmonization, the distributions were similar (as shown in Supplementary Figure 2) and there was no significant differences in the values, *t*(75) = 0.25, *p* = 0.80.


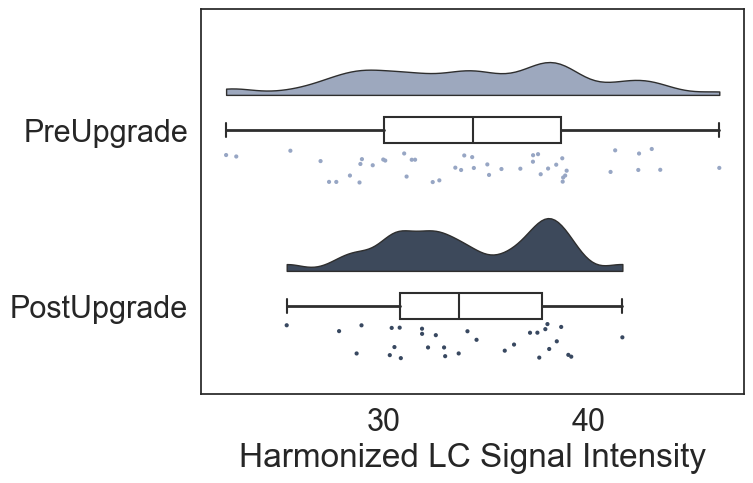


*Supplementary Figure 2. Post-Harmonization LC Signal Intensity values by scanner status (Pre-Upgrade: 3T General Electric MR750 Discovery scanner, Post-Upgrade: a 3T General Electric Signa Ultra High-Performance MR scanner)*

# Pre-Upgrade Results

## Demographics

Pre-upgrade, 23 participants with fibromyalgia (age = 48.39, SD = 9.17) and 23 healthy controls (age = 47.96, SD = 10.15) had valid LC data.

## LC signal and Age

We examined the relationship between age and LC signal. There was a significant correlation between age and LC signal in the healthy control participants (*r*(23) = 0.46, *p* = 0.03) which was not present in the participants with fibromyalgia (*r*(23) = 0.30, *p* = 0.16). Across the pre-upgrade sample, there was a positive association between age and LC signal intensity across all participants (*r*(46) = 0.38, *p* = 0.01). Given these relationships, partial correlations were used to control for age for all other correlations.

## LC signal - Group differences

There were no group differences in the average signal of the central LC (*t*(44) = -0.92, *p* = 0.61).

## LC signal – Fibromyalgia History

Given the substantial heterogeneity in LC signal, we considered whether diagnostic and symptomatic history influenced LC signal. There was no relationship between time since diagnosis of fibromyalgia and LC signal (Spearman’s *rho*(23) = 0.19, *p* = 0.39) nor time since symptom onset and LC signal intensity (Spearman’s *rho*(22) = 0.26, *p* = 0.25).

## LC signal and Fibromyalgia Symptom Assessments

Partial correlations accounting for age did not show any association between LC signal intensity and FIQR (*r*(20) = 0.11, *p* = 0.63), the Central Sensitization Index (*r*(18) = 0.19, *p* = 0.42), the Insomnia Severity Index (*r*(17) = 0.03, *p* = 0.90), the GAD-7 Anxiety measure (*r*(18) = 0.06, *p* = 0.81) or the QIDS-SR Depression Measure (*r*(19) = 0.09, *p* = 0.70).

Investigating the association of cognition and LC signal, there was no relationship between LC signal and the Cognitive Failures Questionnaire (*r*(18) = 0.13, *p* = 0.58) among fibromyalgia participants, controlling for age. When investigating cognitive performance as measured by the THINC-it, controlling for age, there were no associations between LC signal and the “Spotter” measure of attention (*r*(17) = 0.38, *p* = 0.10), the “Symbol Check” working memory measure (*r*(18) = -0.17, *p* = 0.47), the “Trails” measure of executive function (*r*(18) = 0.36, *p* = 0.14), the “Codebreaker” measure of multiple cognitive domains (*r*(18) = 0.08, *p* = 0.74) or the ‘Perceived Difficulties Questionnaire’ (PDQ) assessment of subjective cognitive function (*r*(18) = -0.10, *p* = 0.66).

While controlling for age, investigation of average NM contrast with each of the THINC-it measures across the whole pre-upgrade sample revealed no significant associations in the “Spotter” measure of attention (*r*(38) = 0.26, *p* = 0.11), the “Symbol Check” working memory measure (*r*(40) = -0.06, *p* = 0.73), the “Trails” measure of executive function (*r*(40) = -0.11, *p* = 0.50), the “Codebreaker” measure of multiple cognitive domains (*r*(40) = 0.09, *p* = 0.56) or the ‘Perceived Difficulties Questionnaire’ (PDQ) assessment of subjective cognitive function (*r*(40) = -0.17, *p* = 0.28).

# Post-Upgrade Results

## Demographics

Post-upgrade, 18 participants with fibromyalgia (age = 43.39, SD = 10.83) and 13 healthy controls (age = 43.54, SD = 11.96) had valid LC data.

## LC signal and Age

We examined the relationship between age and LC signal. There was no significant association between unharmonized post-upgrade LC signal and age in the participants with fibromyalgia (*r*(18) = -0.21, *p* = 0.41), in the healthy controls (*r(*13) = 0.22, *p* = 0.47), or across the whole sample (*r*(31) = -0.01, *p* = 0.98).

## LC signal - Group differences

There were no group differences in the average signal of the central LC (*t*(29) = -1.11, *p* = 0.28).

## LC signal – Fibromyalgia History

Given the substantial heterogeneity in LC signal, we considered whether diagnostic and symptomatic history influenced LC signal. There was no relationship between time since diagnosis of fibromyalgia and LC signal (*r*(13) = -0.17, *p* = 0.56) nor time since symptom onset and LC signal intensity (*r*(13) = -0.15, *p* = 0.60), while accounting for age.

## LC signal and Fibromyalgia Symptom Assessments

There was a significant difference between groups in measures of assessments, as reported in Table 1, therefore the associations with LC signal and these measures were conducted only within the participants with fibromyalgia. Partial correlations accounting for age did not show any association between LC signal intensity and FIQR (*r*(15) = 0.05, *p* = 0.86), the Central Sensitization Index (*r*(13) = -0.03, *p* = 0.93), the Insomnia Severity Index (*r*(12) = 0.08, *p* = 0.80), the GAD-7 Anxiety measure (*r*(13) = -0.46, *p* = 0.09) or the QIDS-SR Depression Measure (*r*(13) = -0.29, *p* = 0.30).

Investigating the association of cognition and LC signal, there was no relationship between LC signal and the Cognitive Failures Questionnaire (*r*(13) = 0.13, *p* = 0.64) among fibromyalgia participants, controlling for age. When investigating cognitive performance as measured by the THINC-it, controlling for age, there were no associations between LC signal and the “Spotter” measure of attention (*r*(12) = 0.19, *p* = 0.53), the “Symbol Check” working memory measure (*r*(13) = -0.04, *p* = 0.88), the “Trails” measure of executive function (*r*(13) = 0.22, *p* = 0.43), the “Codebreaker” measure of multiple cognitive domains (*r*(13) = -0.27, *p* = 0.33) or the ‘Perceived Difficulties Questionnaire’ (PDQ) assessment of subjective cognitive function (*r*(13) = 0.17, *p* = 0.54).

While controlling for age, investigation of average NM contrast with each of the THINC-it measures across the whole post-upgrade sample revealed no significant associations in the “Spotter” measure of attention (*r*(24) = -0.14, *p* = 0.51), the “Symbol Check” working memory measure (*r*(24) = -0.13, *p* = 0.52), the “Trails” measure of executive function (*r*(24) = 0.22, *p* = 0.28), the “Codebreaker” measure of multiple cognitive domains (*r*(24) = 0.16, *p* = 0.43) or the ‘Perceived Difficulties Questionnaire’ (PDQ) assessment of subjective cognitive function (*r*(24) = -0.06, *p* = 0.78).
